# Lessons from Randomised Clinical Trials for Triiodothyronine Treatment of Hypothyroidism: Have They Achieved Their Objectives?

**DOI:** 10.1155/2018/3239197

**Published:** 2018-07-16

**Authors:** Rudolf Hoermann, John E. M. Midgley, Rolf Larisch, Johannes W. Dietrich

**Affiliations:** ^1^Department for Nuclear Medicine, Klinikum Lüdenscheid, Paulmannshöher Str. 14, 58515 Lüdenscheid, Germany; ^2^North Lakes Clinical, 20 Wheatley Avenue, Ilkley LS29 8PT, UK; ^3^Medical Department I, Endocrinology and Diabetology, Bergmannsheil University Hospitals, Ruhr University of Bochum, Buerkle-de-la-Camp-Platz 1, D-44789 Bochum, Germany; ^4^Ruhr Center for Rare Diseases (CeSER), Ruhr University of Bochum and Witten/Herdecke University, Alexandrinenstr. 5, D-44791 Bochum, Germany

## Abstract

Randomised controlled trials are deemed to be the strongest class of evidence in evidence-based medicine. Failure of trials to prove superiority of T3/T4 combination therapy over standard LT4 monotherapy has greatly influenced guidelines, while not resolving the ongoing debate. Novel studies have recently produced more evidence from the examination of homeostatic equilibria in humans and experimental treatment protocols in animals. This has exacerbated a serious disagreement with evidence from the clinical trials. We contrasted the weight of statistical evidence against strong physiological counterarguments. Revisiting this controversy, we identify areas of improvement for trial design related to validation and sensitivity of QoL instruments, patient selection, statistical power, collider stratification bias, and response heterogeneity to treatment. Given the high individuality expressed by thyroid hormones, their interrelationships, and shifted comfort zones, the response to LT4 treatment produces a statistical amalgamation bias (Simpson's paradox), which has a key influence on interpretation. In addition to drug efficacy, as tested by RCTs, efficiency in clinical practice and safety profiles requires reevaluation. Accordingly, results from RCTs remain ambiguous and should therefore not prevail over physiologically based counterarguments. In giving more weight to other forms of valid evidence which contradict key assumptions of historic trials, current treatment options should remain open and rely on personalised biochemical treatment targets. Optimal treatment choices should be guided by strict requirements of organizations such as the FDA, demanding treatment effects to be estimated under actual conditions of use. Various improvements in design and analysis are recommended for future randomised controlled T3/T4 combination trials.

## 1. Introduction

Levothyroxine (LT4) substitution remains the standard treatment for patients with hypothyroidism [1]. The evidence base has been repeatedly reviewed by experts and authors of various guidelines [2–6]. Alternative treatments such as T3/T4 combination therapy have not been proven to be a superior option to LT4 monotherapy in randomised controlled trials (RCTs) including meta-analyses comparing the two different modes of treatment [7]. However, this has not defused the situation, which continues to be one of the major controversies in thyroidology.

A disturbing fact has been the existence of a substantial number of patients with persistent symptoms on LT4 treatment. Complaints have been expressed in various forms on the internet and in surveys and have been substantiated by more rigorous retrospective and prospective studies [8–11]. Where subjective dissatisfaction with the received treatment has been acknowledged, opinions are divided about the cause. Nonthyroidal influences such as living with a chronic disease, the burden of a debilitating or malignant disease, the autoimmune process, or obesity have frequently been cited [12–16]. Others have proposed dose inadequacy of LT4 rather than treatment modality to play an important role [2]. The European Thyroid Association (ETA) has issued guidelines supporting combination therapy for patients with persistent symptoms as a valid option to clinicians, but only on an experimental and closely monitored basis [12]. Mirroring the disagreement about the reference range for TSH, the controversy about optimum treatment has been exhaustively reviewed, but not resolved for a decade [17].

## 2. A Fresh Look at the Old Controversy

This raises the question whether there are any newer considerations that may contribute toward a resolution, which is clearly in the interest of patients and clinicians. Although no more RCTs have been recently conducted on the subject, novel insights have emerged from the examination of homeostatic equilibria and experimental treatment protocols in the rodent [19–23]. Generally, if a physiological chain of causal arguments leads up to the final clinical proof, issues can be resolved in a satisfactory way. This is however not the case with T3/T4 therapy. On the contrary, disagreements have widened between the supreme category of evidence in evidence-based medicine (EBM), which can only be derived from RCTs, and other experimental findings.

At least three physiological phenomena challenge the RCT-derived evidence. (1) FT3 concentrations tend to be invariantly lower in LT4-treated patients particularly in athyreotic patients where in the absence of a thyroid gland conversion of T3 from T4 is inefficient, compared to the healthy subject [23]. (2) Experiments performed some 20 years ago in rodents [24], when recently repeated [20], demonstrated that it may be virtually impossible for LT4 treatment alone to restore euthyroidism at the level of various tissues, an endpoint which cannot be readily studied in humans. (3) Thyroid regulation in LT4-treated patients, interrelationships, and homeostatic equilibria between FT3, FT4, and TSH differ from those in healthy subjects [25]. Confirming subjective dissatisfaction with LT4 treatment, hypothyroid patients treated with standard LT4 monotherapy failed in carefully conducted prospective clinical trials to restore their quality of life to a level observed in the healthy population [10].

These contradicting-rather-than-complementing results from different classes of evidence cause a major problem for clinicians and patients alike. Therefore, should we go so far in the formal pursuit of EBM guidelines to insist that RCTs should remain unchallenged and must prevail over physiologically based counterarguments? Is there an important gap in this logic?

## 3. Lessons Learned from RCTs on Combination Treatment

Historic RCTs on T3/T4 treatment have been subjected to meta-analysis and exhaustively reviewed [1–9, 12, 17]. Their inability to prove superiority of the addition of a T3 component to LT4 monotherapy has been used as a strong argument in favour of LT4 standard treatment by guidelines [3, 4]. However, this is more a choice of noninferiority, because superiority has not been established for the standard treatment either. Also, no formal clinical trials have been performed to evaluate the current standard treatment against other treatment modalities such as the original use of natural desiccated thyroid extract (NDT) before that decision was made. Only later was one such trial conducted [26]. Considering other physiologically derived evidence, the alternative option of T3/T4 would seem to be equally justified by RCT outcomes. Non-RCT criteria have strongly influenced preference, foremost a perceived fear of increased adverse T3 effects. Unlike the clinically infrequently used LT3, LT4 has a well-documented history of use and safety profile. As a drug, LT3 is generally more difficult to manage than the prohormone LT4, due to its higher biological activity, shorter half-life, and other pharmacological characteristics [27, 28]. A few available long-term studies suggest that T3 use may still be safe if administered with the necessary care [29].

Examining the strength of evidence and sources of disagreement with other findings, a number of problems can affect RCTs, related to the sensitivity of the QoL instrument, patient selection, effect sizes, statistical power and sample size, and response heterogeneity.

As for the QoL instrument used, historic trials did not rely on validated and thyroid-specific QoL questionnaires, which only became available more recently [30]. As QoL instruments vary considerably in their ability to detect relevant treatment effects in hypothyroidism and exhibit vast differences in effect sizes for individual items, this is an important issue [30]. Unlike ThyPRO, the SF-36 did not achieve even moderate effect sizes for any of its items in a validation trial by Watt et al. [30]. Asking less relevant and nonspecific questions diminishes the overall discriminatory power of the instrument, even more so when p values are adjusted for multiple testing. Targeted thyroid-specific questions may substitute more successfully for a panel of more widely engineered questions [16]. Strong emphasis should therefore be placed on specificity and relevance of questions, use of validated instruments, documented effect sizes, and mandatory realistic pretrial power estimates.

Estimated trial size using contemporary QoL assessment would be 120 patients to detect a marked change in thyroid function during follow-up from hypothyroidism to euthyroidism, based on two-sided t-test statistics and assuming two balanced groups, a moderate effect size (total score 0.59 [30]), alpha error of 5%, beta error of 20% (power 80%), and attrition rate of 30%. The QoL difference between two treatment modalities, such as LT4 and LT3/LT4 combination, can be expected to be much lower than that for a hypothyroid and euthyroid group. This suggests that many historic trials were underpowered, and careful preselection of patients may be necessary to achieve sufficient power for such trials. Post hoc subgroup analysis of patients dissatisfied with LT4 monotherapy, which has been reported in some studies, is statistically controversial and cannot substitute for randomised controlled treatment allocation [31].

Another potential source of loss of statistical power in trials involving the combined administration of T3/T4 relates to widely varying T3 conversion rates among both disease entities and individual subjects [19, 32]. A minor fraction of the peripheral T3 is thyroid-derived, but this amount varies due to the action of a regulatory TSH-T3 shunt [33]. Deiodination capacity is low in patients lacking functional thyroid tissue, e.g., after thyroidectomy, and may decline even further after initiation of LT4 treatment [23, 32]. Effects of adding on T3 may therefore be expected to be more pronounced and beneficial to patients with low T3 concentrations than to those with a higher endogenous or LT4-derived T3 supply. Aggregating patients with various aetiologies and stages of hypothyroidism and different treatment requirements in a single trial may render the expected statistical power of a substitution trial unpredictable [11, 34, 35]. From a clinical point of view, at least patients suffering from autoimmune thyroiditis and thyroid carcinoma should be treated as separate entities.

Symptom rates prior to the trial may give an indication if any improvement could be expected during the trial, irrespective of treatment modality. Averaged scores are less informative in this respect, and averaging techniques also tend to obscure occasions with low occurrence [34]. For symptoms at trial end, the question is how much improvement has occurred. However, a healthy control group was lacking in virtually all combination trials. Neither dose adequacy for LT4 nor LT3/LT4 has been ascertained in the T3/T4 trials. It remains therefore unclear how much either treatment option was able to raise QoL outcomes, compared to a healthy population. Only a control group defining the target level is able to ascertain adequacy of treatment outcome, protecting against an ineffective trial where neither treatment was properly administered, a real possibility indicated by recent prospective studies [10]. The trial must include a control group to address this issue adequately, because within-group comparisons over time are subject to regression to the mean, autocorrelation, and other statistical fallacies.

Inconsistencies between individual trials were assessed in a meta-analysis, although not specifically reported [7]. However, collective analysis may not compensate for shortcomings at the level of several single trials as design errors may distort outcomes in different directions and gravely reduce the legitimacy of an overall conclusion. When evaluating the trustworthiness of the bulk of systematic reviews and meta-analyses, a group of prominent authors and editors came to a devastating judgment and stated that “only about 3% of them are both well done and clinically useful” [35].

The unstratified inclusion of a heterogeneous mix of patients in the RCTs with different aetiologies of the disease, baseline biochemistry, and residual symptoms raises a concern of collider stratification bias [34, 35]. Physiologically, the biochemical equilibria between FT3, FT4, and TSH have been shown to be markedly altered by LT4-treatment, depending on disease aetiology, presence of residual thyroid tissue, and LT4 dose [19, 25, 32]. Treatment responses to LT4 may vary widely between patients even when the recorded biochemical parameter values are similar [11, 36]. This may result in undertreatment of a substantial fraction of patients, because, as reviewed elsewhere, TSH-guided treatment may be compromised in certain circumstances [37]. A prospective study in patients with thyroid carcinoma, measuring biochemical surrogate markers of thyroid-influenced organ function, concluded that LT4-doses needed to be high enough to mildly suppress TSH levels in order to achieve satisfactory treatment outcomes close to euthyroidism, whereas patients with “normal” TSH levels remained mildly hypothyroid [38].

The role of T3 can be briefly expanded in this context. While uncorrelated with TSH in a healthy population over the euthyroid range, mainly due to a physiological TSH-FT3 shunt acting as a balancing tool, FT3 becomes strongly correlated in LT4-treated athyreotic patients lacking this modulatory device [23, 25]. Patients without a thyroid gland suffer a substantial loss of T3 conversion after thyroidectomy, on average approx. 25% in the same patient, compared to the rate prior to surgery [11, 37]. Low FT3 levels, in turn, were associated with persistent complaints in these patients [11]. Theoretical models suggest that the seemingly minor thyroid-derived T3 component has a more important physiological role in stabilising the system than merely directly contributing to the T3 pool [25, 33]. The symptom-FT3-FT4-TSH correlation chain may therefore vary widely, even on occasion inverting correlations.

As for trial design, amalgamation bias (Simpson's paradox [39]) arises when including heterogeneous study groups of patients who have different disease aetiologies, settle at different homeostatic equilibria, and display heterogeneous responses to treatment. Limited data are presently available on intraclass correlations (ICCs) and components of variance although this approach is essential for the interpretation of cluster-based studies [34, 35, 39, 40]. In addition, thyroid hormones are known for their high degree of individuality, with a low ratio of the intraindividual to interindividual variation of approximately 0.5 (low individuality index) [41]. For a person, their perceived “comfort zone” of response, like the intraindividual reference range, is also narrower than for the entire group, as evidenced by substantial intraclass correlation (0.3-0.5) between FT3 and patient complaints during follow-up of patients with thyroid carcinoma [11].

**Figure 1:**
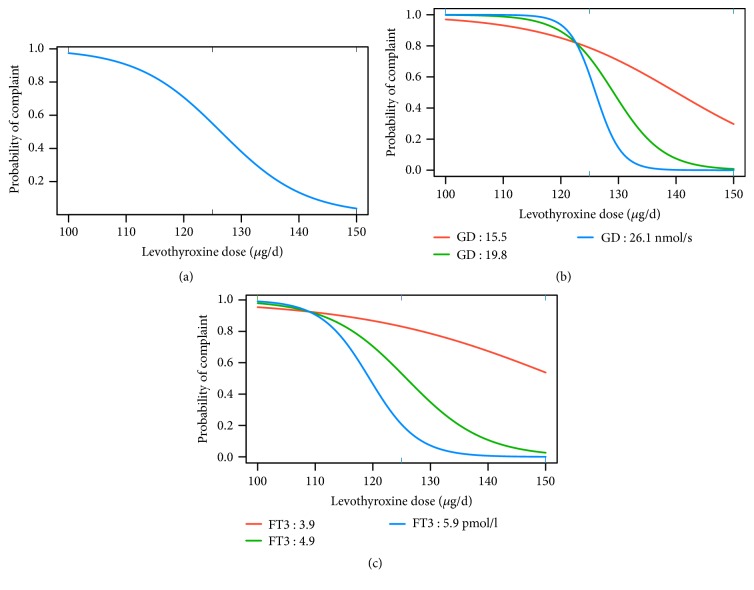
Demonstration of bias by data amalgamation in predicted probability of persisting hypothyroid complaints in response to LT4 dose changes depicted by a simulated trial. Group-level effect (a) is not predictive of individual responses with shifted response curves between patients displaying varying conversion rates (GD (b)), and FT3 concentrations (c). Variable patient individuality of response may impact the averaged outcome in a trial and should be accounted for in the analysis. For the purpose of this demonstration, complementing a study, which has been reported elsewhere [11], we resampled 60 follow-up visits from patients who reported relief of former complaints after LT4 dose increase within a range of 100 to 150 *μ*g/d to generate the structure of an extended generalised linear mixed-effects model [18]. GD refers to global deiodinase activity, a measure of T4 to T3 conversion similar to the molar T3-T4 ratio [11].

Consequently, multiple measures obtained from each subject are nested within that subject. Classical (single‐level) methods cause issues related to the disaggregation of within-person and between-person effects over time, flattening the secondary level, and destroying the interrelationships within it. This is even further exacerbated by the fact that these two levels of influence can operate simultaneously and even in opposite directions [36, 39, 40]. This situation requires a multileveled instead of averaged statistical approach [42]. Personalised treatment targets may therefore be more appropriate than wider range considerations, especially for TSH [36]. In Figure 1, we demonstrate potential impact of conversion efficiency and shifted dose response curves on complaint rates. In such a situation, an error is made when applying the group-level effect to the individuals within the groups, and the patient case-mix may sway averaged outcomes in trials [40]. This may also affect statistical power of a trial (Figure 1, [18]), as discussed above. In sufficiently large samples, response heterogeneity to treatment can and should therefore be assessed, e.g., with latent class analysis [18, 39, 40, 42]. A statistical design and method must be chosen for trial analysis to guard against amalgamation bias, e.g., using multilevel models or cross-over designs.

## 4. Treatment Efficiency and Adverse Effects

While well-suited to prove drug efficacy, RCTs are less able to predict the efficiency of a drug in clinical practice [43]. The latter deals with optimised drug use, as to which patients and conditions may benefit most from the intervention. This differs frequently from averaged results obtained under more artificial conditions in the RCT [34, 35, 40, 42]. While efficiency of T3 addition has not been rigorously studied, differential requirements of patients have been strongly suggested by homeostatic criteria. Mixed inclusion criteria and statistical outcome averaging can be expected to conceal differentiated treatment requirements as well as different risk profiles. Misclassification of true thyroid status by sole TSH measurement and dichotomised analysis of a continuous variable is of concern. For instance, the association between hyperthyroidism and atrial fibrillation (AF) is well established [1], but 10-year AF risk correlated with FT4, not TSH concentrations in euthyroid subjects [44]. Few events were reported in this prospective study in thyroid hormone users (12/720), too few for separate analysis [44]. A high set point appears to be associated with a predetermined genetic risk for cardiac vulnerability [44]. We should note that a disintegration of the TSH-FT4 correlation toward the euthyroid range is unsurprising, given that set points are clustered [22, 45]. When assessing therapeutic risk profiles, this has to be taken into account as a confounder (collider stratification bias, Simpson's paradox), irrespective of study design including RCTs [34, 39, 40, 42]. FT3 concentrations, unlike TSH, correlated with heart rate within a more narrowly defined euthyroid TSH range [46]. A recent meta-analysis confirms that TSH measurements alone are unable to detect cardiac vulnerability with sufficient sensitivity and specificity [47]. In a large prospective study of euthyroid patients with AF undergoing catheter ablation, variations in concentration of thyroid hormones, but not TSH, were associated with recurrence of arrhythmias [48]. Importantly, the risk association, while increasing linearly with FT4, was u-shaped for FT3; both high and low FT3 levels were associated with AF recurrence [48]. Possible pathophysiological explanations for this seemingly paradoxical behaviour involve the actions of nonclassical thyroid hormones, such as reverse-T3 and 3,5-T2 [49]. However, it is not known if raising low FT3 levels may improve the cardiac outcome. Both hyperthyroidism and hypothyroidism have also been shown to facilitate induced atrial fibrillation in rats [50]. Similarly, in a large unselected and predominantly euthyroid sample, we found a u-shaped relationship between the presence of anxiety/depression (Hospital Anxiety and Depression Scale) and serum FT3 levels, but no relationship of mood change with TSH and FT4 concentrations [51]. This association was masked when using standard linear logistic regression and only revealed by more advanced statistical methods (generalised linear-quadratic model) [51]. For bone health, the situation is also more complex in LT4-treated patients, with effects of low-TSH and low-FT3/TSH*β*v on bone health opposing each other [52], although osteoporosis is a well-documented risk of hyperthyroidism [1].

Interestingly, patients treated with LT4 tend to display such a vulnerable constellation of relatively increased FT4 and diminished FT3 concentrations together with shifted equilibria for TSH [23]. This is uniquely different from endogenous hyperthyroidism, reminding us of the traditional distinction between hyperthyroidism and thyrotoxicosis.

From statistical principles, it follows that overlooking an unequally distributed confounding variable will bias the estimates of the effect (Simpson's paradox) [34, 35, 39, 53]. Optimised risk/benefit management requires more than a TSH value, namely, careful analysis of big data including all three thyroid hormones TSH, FT3, and FT4 and using modern statistical tools accounting for intraclass correlations. The wider diagnostic and potential therapeutic implications have been addressed elsewhere [36].

Importantly, while evidence-based medicine has traditionally based stronger emphasis on controlled outcomes achieved by RCTs, the treatment effect under actual “conditions of use” is the determinant of approval for a drug by the American Food and Drug Administration (FDA) [54]. RCTs may not actually meet this FDA criterium, as has been elegantly demonstrated by George et al. [55] from a statistical point of view. The very randomisation required to discriminate the treatment effect from other confounding influences may cause RCTs to poorly predict the actual treatment effect under conditions of intended use [55]. In RCTs, unlike under actual conditions of use where patients know about treatment, patients are uncertain whether they are receiving treatment. This introduces a bias, as patient expectations of improvement substantially impact outcomes and effect sizes of the treatment under actual conditions of use. Improvements in trial design (randomisation to randomisation probabilities, R2R) have been suggested to adjust for participant expectancy about receiving treatment and produce treatment estimates closer to those under actual conditions of use, while retaining core strengths of the RCT [55]. This may be particularly important to consider in QoL trials where patient expectations may markedly influence the subjective treatment outcomes.

## 5. A Need for New Functional Disease Markers

While acting as a generally important outcome measure, QoL may not be an ideal marker for a functional disease. Objective markers of hypothyroidism, which truly reflect the functional thyroid status for various organs, would be required to reconcile RCT results in humans with contradictory experimental evidence from animal studies [20, 24, 56]. TSH can at best serve as a marker of pituitary activity [37]. Even in that role, it provides an adaptive and variable control mode rather than a fixed reference point [36]. Furthermore, peripheral tissues differ from regulatory central organs in important ways, among others in enzyme expression of deiodinases and cellular sensitivity to thyroid hormones [57]. In agreement with physiologically altered equilibria between TSH and FT3, as well as TSH and surrogate tissue markers for hypothyroidism, in LT4-treated patients, animals remained hypothyroid after LT4 administration despite achieving normalisation of their TSH concentrations [19–21, 24, 32, 38, 56]. While generally recognised as useful markers, superiority of surrogate markers such as serum concentrations of methylhistidine, ceruloplasmin, copper, and sex hormone binding globulin (SHBG) over TSH and thyroid hormone concentrations has so far been shown in athyreotic patients and other rare disorders of thyroid homeostasis [38, 58]. Genetic fingerprints and mitochondrial RNA species emerged as novel organ markers, although they are not yet ready for clinical use [59, 60]. Nonclassical thyroid hormones such as reverse-T3 and thyronamines, long believed to be bioinactive, are currently reevaluated [61]. 3,5-T2 correlated with atrial remodelling in the presence of low T3 [49]. Calculated parameters of thyroid homeostasis may serve as functional biomarkers, particularly in subclinical thyroid disease or low-T3 and high-T3 syndromes [62]. These markers may help distinguishing scenarios of different pathophysiology and superficially similar endocrine phenotype and guide stratification of subjects in prospective trials.

## 6. Conclusion

Several areas of recommended improvements in trial design have been identified (Table 1), cautioning against uncritical clinical interpretation of outcomes from previous RCTs on T3/T4 combination therapy. Until better evidence becomes available, the reliance on dated RCTs should be questioned, giving more weight to other forms of valid evidence that have accumulated in recent years and contradict key assumptions from these trials. In addition, strict regulations by organizations such as the FDA demand treatment effects to be estimated under actual conditions of use. To achieve this, inherent shortcomings of RCTs, such as the expectancy bias caused by uncertainty about the treatment in RCTs, as opposed to treatment certainty under conditions of use, must be addressed.

These novel developments should encourage appropriately redesigned and methodologically improved trials to evaluate treatment outcomes of T3/T4 combination therapy, compared with standard LT4 treatment. A clinical focus should be on distinct disease strata, such as patients with autoimmune hypothyroidism or with thyroidectomy for malignant disease and a heterogenous treatment response in identifiable subgroups.

## Figures and Tables

**Table 1:** Suggestions for improvement in trial design for randomised controlled T3/T4 combination studies.

**Criterium**	**Issue of Previous Trials **	**Improvement**
QoL instrument	lack of sensitivity and specificity of older methods	use of validated thyroid-specific methods

Detectable effect on QoL	small effect size	moderate effect size

Statistical power	very low	low

Sample size requirement	very large	large

Patient selection	selection bias due to inclusion of heterogenous patient groups by etiology and prognosis	inclusion of homogeneous diagnostic categories, use of stratified randomisation

Proportion of symptomatic patients	dilution of the true effect	randomized controlled designs for subgroups with persistent symptoms

Treatment-related improvement	healthy control group lacking	inclusion of a healthy control group

Dose adequacy	TSH targets may be misguided.	Treatment-related altered equilibria have to be considered.

Response heterogeneity	wide variation in the treatment response	physiologically based categorisation

Specific confounders	T4 to T3 conversion efficiency	identify conversion issues and apply strata

Statistical analysis	presence of unknown hierarchies and latent groups	latent class analysis

Statistical method	amalgamation bias (Simpson's paradox), disaggregation of within-group and between group effects over time	multilevel models, cross-over design

Patient expectancies	expectancy bias from treatment uncertainty in RCTs vs treatment certainty under actual conditions of intended drug use	randomization to randomization probabilities (R2R) adjusting for differences in patient expectancies

Tissue effects	not addressed by RCTs due to lack of differential markers for organ-specific effects	limited usefulness of surrogate markers, requirement for novel markers

Actions of non-classical thyroid hormones	not addressed	improvement of assay technology, evaluation as possible additional treatment targets

Safety profile	not addressed by RCTs	prospective acquisition and analysis of big data, especially from T3 users

Drug-related issues of LT4	generally reduced and variable T4 to T3 conversion rates	measuring conversion efficiency and targeted T3 addition

Drug-related issues of LT3	pharmacological properties, among others short half-life, high peak levels	slow-release preparations

Drug-related issues of natural desiccated thyroid extracts	popular choice among patients, but few studies	effective large-scale trials

## Data Availability

No original data were used for this study, a systematic review. Previously reported original data were reused in part for Figure 1 and are available at [doi:10.1055/s-0043- 125064, doi:10.1371/journal.pone.0187232]. These prior studies and datasets are cited at relevant places within the text as [11, 22]. Data have been deposited according to the process of the respective journal together with the original publication.
